# The First Step Toward Diagnosing Female Genital Schistosomiasis by Computer Image Analysis

**DOI:** 10.4269/ajtmh.15-0071

**Published:** 2015-07-08

**Authors:** Sigve Dhondup Holmen, Elisabeth Kleppa, Kristine Lillebø, Pavitra Pillay, Lisette van Lieshout, Myra Taylor, Fritz Albregtsen, Birgitte Jyding Vennervald, Mathias Onsrud, Eyrun Floerecke Kjetland

**Affiliations:** Norwegian Centre for Imported and Tropical Diseases, Oslo University Hospital, Oslo, Norway; Faculty of Medicine, University of Oslo, Oslo, Norway; Department of Microbiology, Haukeland University Hospital, Bergen, Norway; Department of Biomedical and Clinical Technology, Durban University of Technology, Durban, South Africa; Department of Parasitology, Leiden University Medical Center, Leiden, The Netherlands; Discipline of Public Health Medicine, Nelson R. Mandela School of Medicine, University of KwaZulu-Natal, Durban, South Africa; Department of Informatics, University of Oslo, Oslo, Norway; Parasitology and Aquatic Diseases, University of Copenhagen, Copenhagen, Denmark; Department of Gynaecology, Oslo University Hospital, Oslo, Norway

## Abstract

*Schistosoma haematobium* causes female genital schistosomiasis (FGS), which is a poverty-related disease in sub-Saharan Africa. Furthermore, it is co-endemic with human immunodeficiency virus (HIV), and biopsies from genital lesions may expose the individual to increased risk of HIV infection. However, microscopy of urine and hematuria are nonspecific and insensitive predictors of FGS and gynecological investigation requires extensive training. Safe and affordable diagnostic methods are needed. We explore a novel method of diagnosing FGS using computer color analysis of colposcopic images. In a cross-sectional study on young women in an endemic area, we found strong associations between the output from the computer color analysis and both clinical diagnosis (odds ratio [OR] = 5.97, *P* < 0.001) and urine microscopy for schistosomiasis (OR = 3.52, *P* = 0.004). Finally, using latent class statistics, we estimate that the computer color analysis yields a sensitivity of 80.5% and a specificity of 66.2% for the diagnosis of FGS.

## Introduction

Gynecological disease due to *Schistosoma haematobium*, female genital schistosomiasis (FGS), is endemic throughout Africa with a major burden of disease in sub-Saharan Africa.^1^–^3^ It is related to poverty through poor sanitation and dependency on unprotected water sources such as rivers, dams, and lakes.^4^ The populations at risk have limited access to adequate health care.^2^

FGS may create lesions and inflammation in the female genital tract, causing bleeding, pelvic discomfort, dyspareunia, and infertility.^3^,^5^ Furthermore, there is growing concern that it may also increase the risk of acquiring human immunodeficiency virus (HIV).^6^–^10^ The relationship between FGS and HIV has been explored in two independent cross-sectional studies including (in total) almost 1,000 women, showing that women with urogenital schistosomiasis have a higher prevalence of HIV (increased odds ratio [OR] of 2.9–4.0).^7^,^8^

The lesions associated with FGS are thought to be due to the parasite's deposition of ova in the genital mucosa.^11^ They may appear as singular grains, grains in clusters, and as homogenous, yellow areas.^12^ Furthermore, characteristic blood vessels in the cervical mucosa are an important sign of genital schistosomiasis.^13^

In HIV endemic areas where women cannot choose to abstain from intercourse, crush biopsies should only be taken directly from lesions if sexual abstinence or condom protection can be assured 1 day prior to, and 14 days following the procedure.^5^,^14^ Moreover, pathology services are scarce and transport of specimens (and patients for return visits) are often impossible. A safe, affordable, and reliable diagnostic tool has yet to be developed.^5^

It is estimated that more than 60% of the sub-Saharan population has access to mobile phones.^15^ In Kenya, mobile banking was used by 73% of bank customers in 2012 and similar initiatives exist or are being established in other African countries.^16^ In rural areas where specialized health care is not available, telemedicine is currently in use for follow-up of antiretroviral treatment of HIV and in the fields of pathology, dermatology, and radiology.^17^ Likewise, lesions characteristic of FGS have features that could be suitable for telemedical diagnosis. In this study, we seek to explore if a recently published method of computerized color detection may be used to detect FGS.^18^ If so, it would represent an affordable alternative to laboratory-based techniques and it may overcome the price barrier against investing in colposcopic equipment.^19^

## Materials and Methods

### Study area and population.

The study area was situated along the coast of KwaZulu-Natal, South Africa and had a population of 710,000 with 51% below the age of 20 years and 55% females. According to the South African antenatal sentinel survey of 2011, 37% of women attending antenatal care in KwaZulu-Natal were HIV-positive and in the district where this study was conducted, the prevalence was reported to be 42%. The most recent (2009–2010) survey on urogenital schistosomiasis in this district reported a 32% prevalence of *S. haematobium* egg patent infection as diagnosed by microscopy among children in primary school.^20^ According to the South African census of 2007, almost one-third of the households in this area did not have access to piped water in their community, 59% of households used a pit latrine, and 9% did not have any toilet facilities.

### Study design and participants.

**Figure 1.**
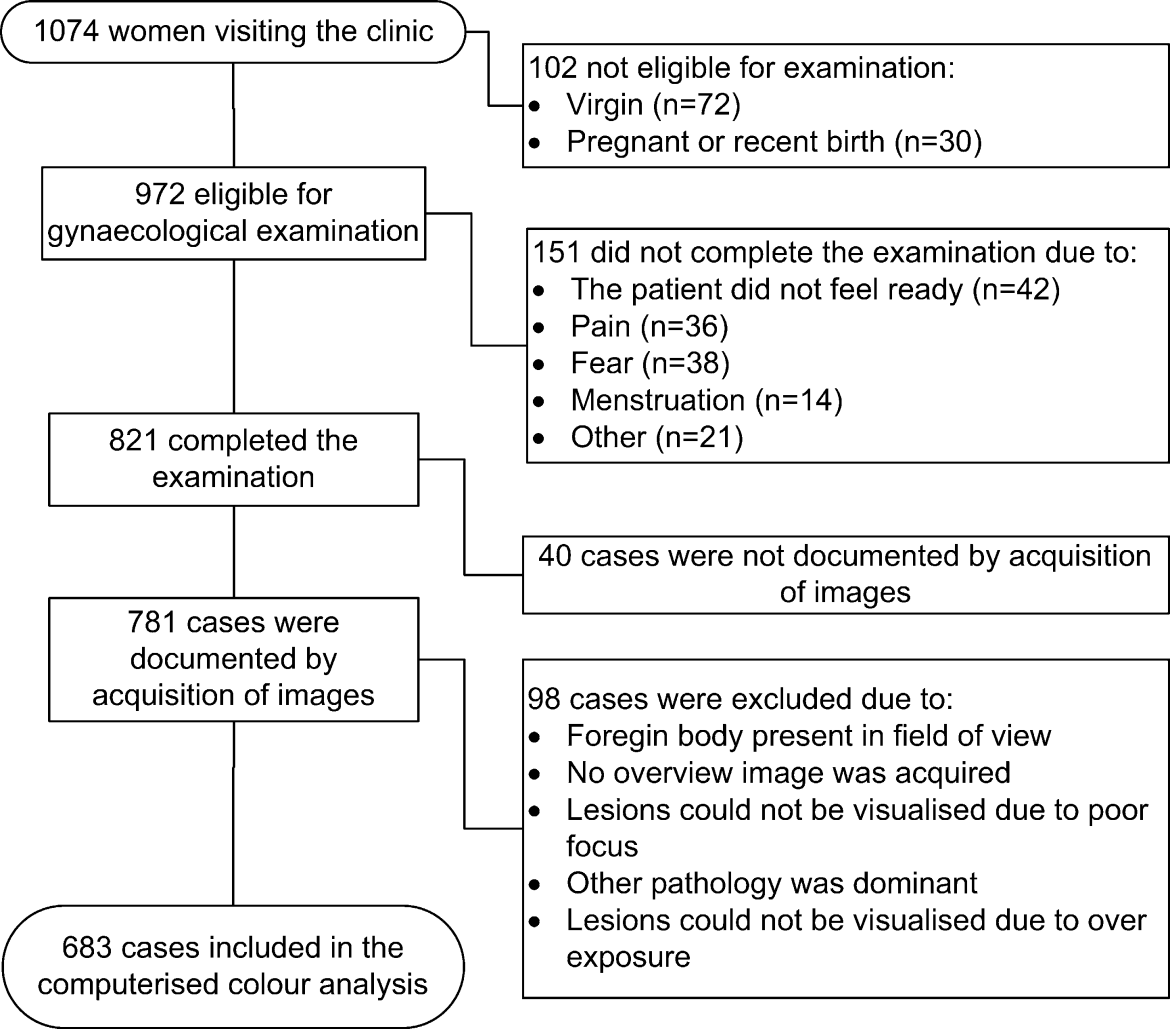
Selection of image material.

This was a cross-sectional study nested in a larger, school-based survey on FGS in women aged 16 and above. In 2011 and 2012, we included all sexually active, non-pregnant young women who had not recently given birth and who consented to a comprehensive gynecological examination with digital documentation (unpublished data). Recruitment was not based on symptoms or test results. The participants were recruited continuously, interrupted by school exams and holidays (Figure 1).

For the computer color analysis, we manually searched the documentation database for images that fulfilled the following criteria: the image had not been part of the training set used in the development of the method^18^ and the cervix should be in the field of view. The sandy patch (if present) should be located on the cervical mucosa and visible in the image (but not necessarily the central element). There should be no foreign material in the field of view (swab, spatula, acetic acid, etc.) and the exposure and focus should be adequate for visualization of the lesion, though not necessarily perfect.

### Image material and clinical investigation.

The lesions associated with FGS are often referred to as sandy patches because of their yellow, sometimes grainy, appearance. It is believed that the grains represent schistosome ova, possibly calcified and/or surrounded by immunologic cells. They have a characteristic rice grain shape, the size has been estimated to measure 0.05 × 0.2 mm.^13^ The sandy patches appearing without visible grains at 15× magnification are also strongly associated with FGS but their diagnostic value is less certain.^13^ The color is that of the grainy sandy patches.

An FGS consensus meeting held in Copenhagen, October 2010 followed by a meeting in Durban, January 2013, considered clinical and laboratory results from several African studies.^5^ The meetings concluded that, in patients from areas endemic of *S. haematobium*, one of the following three colposcopic findings together with one laboratory test, may serve as an adequate diagnosis for genital schistosomiasis in research: 1) sandy patches appearing as single grains or in clusters, 2) sandy patches appearing as homogenous, yellow areas, or 3) rubbery papules.

The women in the study were examined using an autoclaved metal speculum and an Olympus OCS 500 colposcope with a mounted Olympus E420 10 megapixel (Mpx) single-lens reflex (SLR) camera (Olympus Corporation, Tokyo, Japan) or a Leisegang colposcope with a Canon EOS 40D 10 Mpx SLR (Canon Inc., Tokyo, Japan). The image files were stored using high-quality JPEG compression (the most common file format used in digital cameras). Lesion size, appearance, and location were recorded by digital sketching on a simplified model of the cervix. In a subsample, after lesions had been identified by colposcopy, we attempted to revisualize lesions using flashlights and a 5× magnifying glass, all available in local convenience stores. Furthermore, a high-end flashlight available in airports (Maglite, MAG Instrument, Ontario, CA) was tested.

The fact that the sandy patches appear more yellow than the surrounding mucosa, allowed us to develop a method in which the color properties of the sandy patches are used for detection.^18^ This has been adapted into an automated analysis of colposcopic images.

### Image analyses.

**Figure 2.**
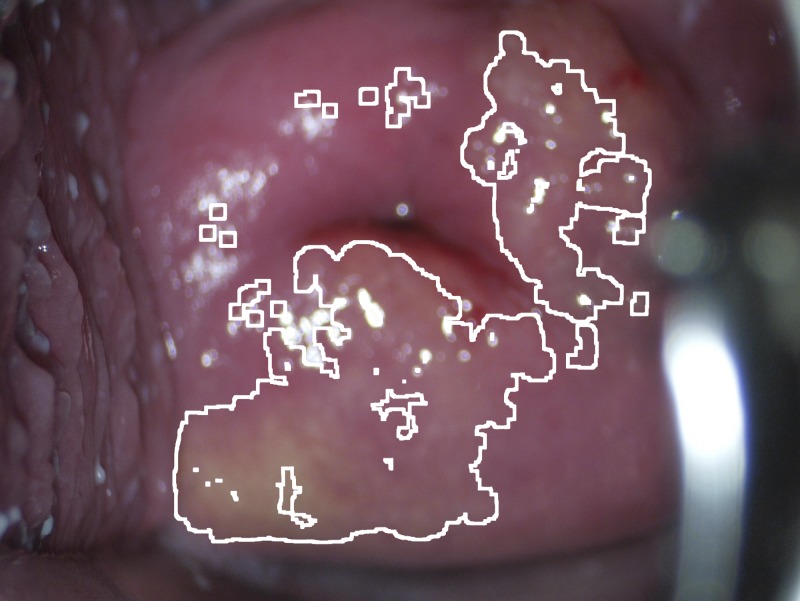
Sandy patches on a cervix detected by color analysis. The computer color analysis creates a demarcating line for investigator verification of the result. The white lines show the borders between pathology (the sandy patch color) and normal mucosa.

The characteristic color properties of the yellow sandy patches and the computer color analysis have been described in detail previously.^18^ In brief, it identifies the region of interest (the ectocervical mucosa) and splits the image in multiple color channels: red, green, blue, hue, saturation, and yellow/blue. The color range (relative to the mucosal color) corresponding to the “typical” sandy patch color was established by measurements on a training set. The mean color value of the mucosa is measured in each image to compensate for varying exposure and color balance, thus representing an adaptive color analysis which can perform well in a wide range of conditions. For every color channel, clusters of pixels that match the predefined sandy patch color range (relatively more yellow than the surrounding mucosa) are detected. Finally, the intersection of detected pixels in all the color channels is calculated, so that only pixels matching in all the color channels are kept. The final output is a numerical value, which corresponds to the combined area of detected pixels (expressed as millions of pixels, Mpx), which in turn can be used to define a cut-off value for a positive diagnosis and to measure the lesion size. Furthermore, the detected pixels may be overlayed on the original image to allow for visual quality control (Figure 2).

### Laboratory analyses.

Urine microscopy for schistosome ova was performed on a single urine sample collected between 10 am and 2 pm on the day of clinical examination. Every urine sample was analyzed by two independent technicians.^21^

Samples of cervico-vaginal lavage (CVL) were obtained by spraying 10 mL of saline onto the ectocervical mucosa followed by withdrawal back into a syringe. Lavage and urine samples were analyzed using an in-house real-time polymerase chain reaction (PCR) technique for detection of schistosome DNA (Leiden University Medical Center, Leiden, The Netherlands).^22^ Furthermore, the CVL was analyzed for possible confounding factors: *Herpes simplex* virus and *Trichomonas vaginalis* were analyzed using in-house PCR tests (Laboratory of Infection, Prevention and Control, University of KwaZulu-Natal, Durban, South Africa). *Chlamydia trachomatis* and *Neisseria gonorrhoea* were analyzed using strand displacement assay on the ProbeTec machine (Becton, Dickinson and Company [BD], Franklin Lakes, NJ). *Treponema pallidum* was analyzed using rapid plasma reagin on serum (Macro Vue 110, BD, Franklin Lakes, NJ).

### Ethical considerations.

The study was granted permissions by four ethics committees: the European Group on Ethics in Science and New Technologies in 2011 (Ref IRSES-2010:269245); the Biomedical Research Ethics Administration, University of KwaZulu-Natal on February 20, 2011 (Ref BF029/07); the Department of Health, Pietermaritzburg, KwaZulu-Natal on February 3, 2009 (Ref HRKM010-08); and the Norwegian ethics committee of Eastern Norway on September 17, 2007 (Ref 469-07066a1.2007.535). The Departments of Health and Education in Ugu district, KwaZulu-Natal also gave permissions.

We obtained written informed consent from all participating women. They were informed of the right to withdraw from the study and examinations at any time. All investigating clinicians were female. The participants were informed that specimens were to be tested for HIV and other sexually transmitted infections (STIs). We provided pre- and post-HIV-test counseling. Test results were conveyed to participants unless they did not wish to know. All participants were offered treatment or referral for conditions that were diagnosed during the investigations. All colposcopic images are nonidentifiable, they only depict the uterine cervix and contain no names.

### Statistical analyses.

Statistical analyses and graphs were produced using IBM SPSS Statistics Version 19 (IBM Company, Chicago, IL). Uni- and multivariable logistic regression analyses were performed, using the clinical diagnosis, urine and CVL analyses results as independent variables with the output of the computer image analysis as a continuous variable (in units of 1 Mpx) as well as possible confounding factors as covariates. Furthermore, we used logistic regression with mean urine prevalence in the schools as the independent variable and presence of sandy patches by clinical inspection or by computer color analysis as the dependent variable (ORs were given for a 10% increase in school prevalence).

The possible confounding factors (STIs) were analyzed using linear regression with the image analysis' output in pixels as dependent variable. Any significant confounder would be included in the multivariable models. A significance level of 5% was used throughout.

Latent class statistics were applied using urine microscopy for schistosome ova, clinical investigation for sandy patches, *Schistosoma* PCR of urine and CVL, school prevalence by urine microscopy, and the output from the computer analysis. Competing models with one to five latent classes were constructed and the optimal model was selected based on parsimony, maximum likelihood, and the Akaike and Bayesian information criterions. All models were “unconstrained” in that we did not make any assumptions (constraints) on sensitivity and specificity regarding any of the included variables. All latent class statistics were calculated using STATA (StataCorp, College Station, TX) with the LCA STATA plugin (The Methodology Center, Penn State, PA). It allows for the analysis of multiple variables that imperfectly measure a condition of which the true status is unknown. The status is referred to as the latent variable, and consists of two or more classes.^23^,^24^ The performance of the computer color analysis was compared with the latent class (serving as surrogate gold standard) by plotting a receiver operating characteristics (ROC) curve and measuring the area under the curve (AUC). The optimal cut-off level (in number of pixels) for defining a positive case was found by identifying the point on the ROC curve closest to the upper left corner.

## Results

As shown in Figure 1, 1,074 women from 57 schools were invited for gynecological examinations. Of the 821 investigated women, the clinicians indicated the presence of sandy patches in 26% (*N* = 212). Sandy patches appearing as grains were described in 112 (14%) participants and sandy patches appearing as homogenous, yellow areas were described in 128 (16%). Presence of both lesion types was described in 28 (3%). The laboratory findings in urine and CVL are presented in Table 1.

Using a flashlight and 5× magnifying glass on positive cases, it was possible to visualize the lesions in 4 of 72 tested cases with FGS (5.6%), applying the high-quality flashlight after the low-quality flashlight.

The output from the computer color analysis was not associated with any of the STI results: *N. gonorrhoea* (*P* = 0.27), *Treponema pallidum* (*P* = 0.40*), C. trachomatis* (*P* = 0.82), *H. simplex* (*P* = 0.42), *Trichomonas vaginalis* (*P* = 0.55), *Haemophilus ducreyi* (*P* = 0.70), and HIV (*P* = 0.34). Neither of these was therefore included in the multivariable models.

We found a strong association between the output from the computer color analysis and a colposcopy diagnosis of FGS (OR = 5.97, 95% confidence interval [CI] = 2.63–13.55, *P* < 0.001), which remained significant when adjusting for age in a multivariable analysis (*P* < 0.001). Furthermore, the computer color analysis was associated with finding *S. haematobium* ova in urine by microscopy (OR = 3.52, 95% CI = 1.50–8.27, *P* = 0.004), and by PCR of urine (OR = 2.99, 95% CI = 1.20–7.45, *P* = 0.019) but not by PCR of CVL (OR = 2.34, 95% CI = 0.70–7.79, *P* = 0.167).

The mean prevalence of urinary schistosomiasis in the school of the pupil was associated with the finding of sandy patches by clinical investigation (OR = 1.23, 95% CI = 1.078–1.40, *P* = 0.002). However, there was no association with the yellow color detection by computer color analysis (OR = 1.09, 95% CI = 0.96–1.25, *P* = 0.191).

**Figure 3.**
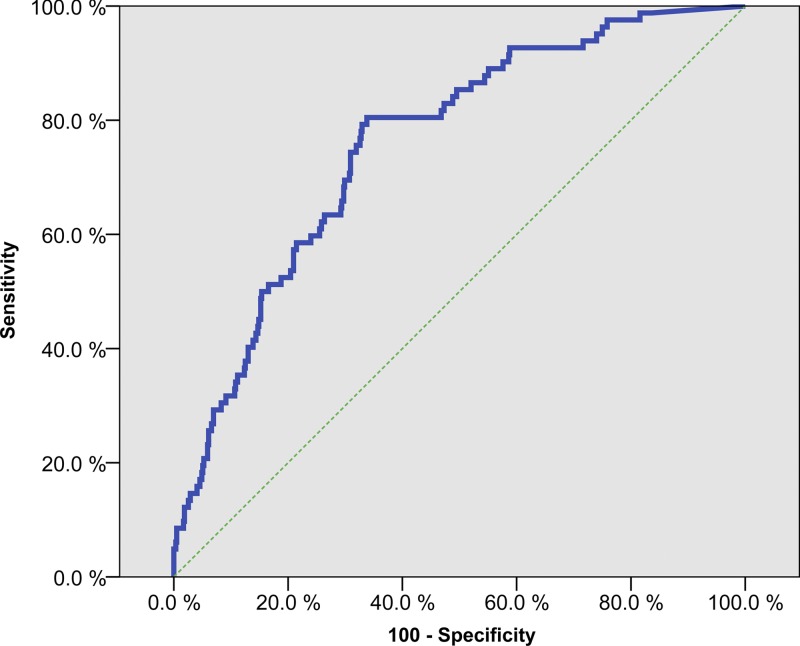
Receiver operating characteristics (ROC) curve of the computer image analysis vs. the latent class chosen as a surrogate gold standard.

Using latent class statistics, we fitted a model dividing the population into three classes. Table 2 shows a contingency table with the probabilities of having a positive finding for an observed variable, conditional on each of the three latent classes: 1) The first class, which represented 80% of the population, was characterized by having no particular findings in any of the observed variables. This class was labeled “Schistosomiasis negative.” 2) Individuals in the second class were found to have high risk for positive urine microscopy and PCR for presence of schistosome ova as well as attending a school with medium–high or high prevalence of schistosomiasis (combined risk of 82%). However, they had very low risk of positive findings by clinical investigation and computer color analysis. This class, which represented 6% of the population, was labeled “Urinary schistosomiasis without FGS.” 3) The final, third class, which represented 14% of the population, was characterized by individuals who were likely to have positive findings in urine (both microscopy and PCR) as well as by computer analysis. In addition, they had a 46% chance of having a positive finding by clinical investigation and a 40% chance of having a positive PCR result in CVL. Furthermore, the combined risk of attending a medium–high or high prevalence school was 78%. This class was labeled “FGS positive with eggs in urine or lavage.” This final class was used as a surrogate gold standard to estimate the predictive quality of the computer color analysis. Sensitivity was calculated to 80.5% and specificity 66.2% using the optimal cut-off level for defining a positive diagnosis, as determined by using the ROC curve (Figure 3

, AUC = 0.76, 95% CI = 0.71–0.81, *P* < 0.001).

## Discussion

In this study, we found that computer color analysis may be of diagnostic value as a tool for objective detection of lesions caused by FGS with a sensitivity of 80.5% and specificity of 66.2% in colposcopic images.

### Clinical relevance.

Diagnosing FGS is an unresolved problem for which a safe, reliable, and affordable solution is long overdue.^5^ The diagnostic challenges lie partly in the nature of the lesions. The calcified (dead) ova may persist after the live parasites have been eradicated and the inflammatory processes surrounding the ova may still persist. It has been shown that genital mucosa with calcified ova harbor a higher density of CD4^+^ T-lymphocytes, creating a setting that may increase the risk of HIV infection.^5^,^9^ Furthermore, these lesions have been found to persist after standard and high-dose treatment with praziquantel.^13^ Therefore, correct diagnosis may help in identifying patients who are susceptible to HIV infection.

Techniques that identify only the presence of live ova or schistosome DNA in vaginal excretions will not detect calcified ova that may still be the cause of severe morbidity. This may partly explain why PCR in CVL does not detect all the cases in which sandy patches are seen. In a previous study, it was found that PCR of CVL detected 56% of cases with clinical findings of sandy patches, which corresponds well with our data.^25^ We found that the flashlight and magnifying glass was not useful in diagnosis. Microscopy of wet smears and Pap smear have likewise been found to have sensitivities below 15%.^11^,^13^ Analyses of urine (dipstick, microscopy, PCR, and antigen detection) may be used as indications of urinary schistosomiasis, but have poor predictive value for genital morbidity.^26^–^28^ Furthermore, the standard field-applicable test of urine microscopy has low sensitivity, especially in low-endemic areas and repeated samples should be acquired when possible.^3^ In our study, we were only able to collect a single urine sample. Serologic methods detecting schistosome antibodies can neither distinguish between current and past infection, nor between FGS and other manifestations of schistosomiasis.^29^ Current and past infection may be distinguished by the detection of circulating anodic antigens in urine but the test neither provides any information on the site of morbidity nor of the chronic, post-parasite manifestations.^30^

In this study, we identified three classes by LCA, one of which represents positive urinary findings without genital lesions. Similarly, one could expect to find a class with genital lesions but without detectable eggs in urine or lavage. Although we attempted to identify such a “fourth class,” the models were clearly inferior to the three-class model in terms of model fit and information criterions. This may be due to the fact that the variables used to construct the model did not contain information on calcified ova and visual inspection by colposcopy cannot distinguish between viable and calcified ova. An ideal validation of this new method would have been performed using lesion biopsies as the gold standard. However, most areas endemic of schistosomiasis are also endemic for HIV, rendering such a study difficult without exposing participants to unnecessary risk of infection.^10^

Clinical diagnosis of schistosomiasis by visual inspection using a colposcope has been deemed a safe and acceptable approach.^5^ However, a study performed in five sub-Saharan countries in 2001, showed that colposcopes were not available in any of the district hospitals, and in only 6% of provincial hospitals.^19^ Furthermore, colposcopic equipment is expensive with the cheapest models costing around $2,000. It is therefore necessary to find alternative means of diagnosis that do not require a colposcope or a medical expert at the point of care. The method that we propose in this study, using computer color analysis, represents the first step in developing a tool for safe and objective assessment of the lesions as an aid in a clinical diagnosis, possibly in combination with image analysis for cancer diagnosis.^31^,^32^

The method is ideal for use in remote areas with limited access to electricity and infrastructure. It has been shown that images acquired with a cell phone camera were adequate in visualizing the cervix for evaluation of pre-cancerous, gynecological lesions.^33^ By using simple electronic devices such as handheld cameras, mobile phone cameras, or other, there will be no need for expensive investments in new equipment. There are a number of cancer-screening programs in sub-Saharan Africa where small handheld cameras are currently used for documenting findings after application of acetic acid visual inspection with acetic acid (VIA) instead of using a colposcope.^34^,^35^ Where such equipment is already in use, implementing new, software-based analyses comes at no additional cost. Together with standardized protocols for acquiring the images, this tool might even be used for quantification of the lesion size to monitor treatment effect over time.

Furthermore, distribution of a software tool to remote areas would be simple and cheap. If made available online, it could easily be downloaded to imaging devices. Instructions and training material could equally be made available, either bundled in the software or as an online resource. Furthermore, online availability allows for upgrades of the software's diagnostic and management algorithms as scientific progress is made and accuracy improved.

Although the primary intention for this tool is rural use in endemic areas, it is also possible to imagine that it could be of use to clinicians in the western world. Few have experience with the lesions caused by FGS. It may be found in western travelers, but more importantly in immigrants from endemic areas.^36^,^37^ This tool could provide an objective assessment of genital lesions that the clinician may be unfamiliar with.

The image material was acquired using colposcopes and high-resolution SLR cameras. These are unrealistic conditions in many developing countries. Our group has done a preliminary simulation where images were resized and random noise was added to decrease the quality to the equivalent of a high-end mobile phone camera (iPhone 4, Apple, Cupertino, CA; Samsung Galaxy S2, Samsung, Seoul, South Korea). We found that the computer analysis performed almost equally well. However, when more noise was added, the precision was drastically reduced. The analysis should be evaluated for use on images acquired using simple technology, such as mobile phone cameras or other handheld imaging devices.

### Future research.

Although the computer color analysis shows promising results in terms of accuracy, the specificity is still lacking. One reason for the high rate of false positives may be detection of other gynecological conditions, such as nabothian cysts, which may also appear yellow. However, the nabothian cysts have a very smooth appearance as opposed to the sandy patches and we believe that texture analysis should be explored for a more precise classification. Furthermore, abnormal vessels in FGS are described as convoluted (cork screw), reticular, or branched with uneven caliber; and the analysis should be expanded to recognize these.^13^

It is necessary to assess the safety of using alternative electronic devices for diagnostic purposes in FGS. Although the images do not depict identifiable traits in the patient, they should be considered sensitive and as such, they must be encrypted and not stored longer than necessary. It may not even be necessary to store the original image if the computer analysis can be performed directly on the device. In the case, the images needed to be transmitted for telediagnostic purposes, it would be of paramount importance to implement proper encryption technology. This is particularly important to consider if one were to use a mobile phone camera to capture and/or transmit the images. Furthermore, it is necessary to assess the patient acceptability of using alternative imaging devices, such as mobile phone cameras. Patients may not feel comfortable with the use of such devices unless it is made clear how images are processed and protected.

Cervical cell atypia is a very important differential diagnosis that must not be confounded with FGS, as correct follow-up and treatment is critical to patient outcome. Several methods have been published on computerized analysis of cervical images stained with acetic acid for screening of cervical cell atypia.^31^,^32^,^38^ In developing an automated analysis for FGS, it would be very useful to implement a conjoint tool to increase the specificity of the diagnostic advice and avoid fatal misclassification of lesions.

## Conclusion

Computer color analysis may be of diagnostic value as a tool for objective detection of lesions caused by FGS and it can represent an affordable alternative to laboratory and colposcopic techniques.

## Figures and Tables

**Table 1:** Computer and laboratory analyses compared with the clinical finding of sandy patches

Positive test	Sandy patches n/total* (%)	No sandy patches n/total* (%)	*P* value†
Computer color analysis‡	105/183 (57.4%)	186/495 (37.6%)	< 0.001
*Schistosoma* PCR in CVL	25/196 (12.8%)	36/425 (8.5%)	0.110
*Schistosoma* PCR in urine	65/186 (35%)	76/421 (18.1%)	< 0.001
Urine microscopy for *Schistosoma haematobium*	62/206 (30.1%)	87/588 (14.8%)	< 0.001

PCR = polymerase chain reaction; CVL = cervico-vaginal lavage.

* The total varies due to different number of specimens available for analysis.

† χ^2^ test.

‡ Using a cut-off of 0.65 Mpx in defining a positive case.

**Table 2:** Latent class analysis was used to classify the population (*N* = 1,074) in three classes based on all available information

Observed variables	Probability of having a positive variable conditional on class adherence		
	Schistosomiasis negative	Urinary schistosomiasis without FGS	FGS positive with eggs in urine or lavage
Computer color analysis	0.38	0.00	**0.70**
*Schistosoma* PCR in CVL	0.02	0.27	0.40
*Schistosoma* PCR in urine	0.04	**0.89**	**0.88**
Urine microscopy	0.01	**1.00**	**0.74**
Clinical finding of sandy patch	0.09	0.00	0.46
School prevalence 0–9%	0.17	0.00	0.06
School prevalence 10–19%	0.36	0.18	0.17
School prevalence 20–29%	0.34	**0.55**	0.35
School prevalence > 30%	0.14	0.27	0.43

FGS = female genital schistosomiasis; PCR = polymerase chain reaction; CVL = cervico-vaginal lavage.

Probabilities exceeding 0.5 are indicated in bold.
